# Maternal mental health after infant discharge: a quasi-experimental clinical trial of family integrated care versus family-centered care for preterm infants in U.S. NICUs

**DOI:** 10.1186/s12887-023-04211-x

**Published:** 2023-08-10

**Authors:** Linda S. Franck, Caryl L. Gay, Thomas J. Hoffmann, Rebecca M. Kriz, Robin Bisgaard, Diana M. Cormier, Priscilla Joe, Brittany Lothe, Yao Sun

**Affiliations:** 1 Department of Family Health Care Nursing, University of California San Francisco (UCSF), 2 Koret Way, N411F, Box 0606, San Francisco, CA 94143 USA; 2 Department of Epidemiology and Biostatistics, Office of Research, School of Nursing, UCSF, San Francisco, CA USA; 3 Intensive Care Nursery, UCSF Benioff Children’s Hospital, San Francisco, CA USA; 4 NICU and Pediatrics, Community Regional Medical Center, Fresno, CA USA; 5 Division of Neonatology, UCSF Benioff Children’s Hospital, Oakland, CA USA; 6 Will’s Way Foundation, Chicago, IL USA; 7 Division of Neonatology, Department of Pediatrics, UCSF, San Francisco, CA USA

**Keywords:** Family partnerships, Infant, Maternal depression, Perinatal PTSD, Parental stress

## Abstract

**Background:**

Involvement in caregiving and tailored support services may reduce the risk of mental health symptoms for mothers after their preterm infant’s neonatal intensive care unit (NICU) discharge. We aimed to compare Family-Centered Care (FCC) with mobile-enhanced Family-Integrated Care (mFICare) on post-discharge maternal mental health symptoms.

**Method:**

This quasi-experimental study enrolled preterm infant (≤ 33 weeks)/parent dyads from three NICUs into sequential cohorts: FCC or mFICare. We analyzed post-discharge symptoms of perinatal post-traumatic stress disorder (PTSD) and depression using intention-to-treat and per protocol approaches.

**Results:**

178 mothers (89 FCC; 89 mFICare) completed measures. We found no main effect of group assignment. We found an interaction between group and stress, indicating fewer PTSD and depression symptoms among mothers who had higher NICU-related stress and received mFICare, compared with mothers who had high stress and received FCC (PTSD: interaction β=-1.18, 95% CI: -2.10, -0.26; depression: interaction β=-0.76, 95% CI: -1.53, 0.006). Per protocol analyses of mFICare components suggested fewer PTSD and depression symptoms among mothers who had higher NICU stress scores and participated in clinical team rounds and/or group classes, compared with mothers who had high stress and did not participate in rounds or classes.

**Conclusion:**

Overall, post-discharge maternal mental health symptoms did not differ between the mFICare and FCC groups. However, for mothers with high levels of stress during the NICU stay, mFICare was associated with fewer post-discharge PTSD and depression symptoms.

## Introduction

Parents provide essential caregiving support for preterm infants admitted to neonatal intensive care units (NICU) to promote infant growth and development, including breastfeeding, skin-to-skin contact, developmentally supportive care, positive sensory stimulation, pain management and massage [1]. Although many NICUs claim to provide family-centered care (FCC), full parental partnership in NICU care and decision-making is often lacking, and disparities exist in parental involvement related to parental and NICU resources [2, 3]. The slow adoption of evidence-based, parent-partnered, FCC clinical practices suggests that more attention is needed on how care delivery is structured, and new models of care may be required.

Family-Integrated Care (FICare) is a parent-partnered NICU care delivery model that provides well-defined yet flexible protocols for ensuring meaningful participation of parents in all aspects of NICU caregiving, from an individual infant’s bedside to the hospital boardroom. FICare has four main pillars: NICU environment, NICU team education and support, parent education, and parent support [4, 5]. Mobile technology may further promote FICare delivery [6]. Clinical trials and quality improvement evaluations of FICare in high- and middle-income countries, and at all levels of neonatal care, have shown that parents of preterm infants experience lower stress, greater confidence in infant caregiving and improved communication with the healthcare teams when NICUs adopt the FICare model [6–14]. Lower chronic physiological stress at 18 months (child’s corrected age) has also been reported for mothers after discharge from NICUs providing the FICare model compared with those provided FCC [15].

The United States (US) healthcare and social contexts are different than in other countries and these differences may affect NICU implementation of FCC and FICare and parental uptake of supportive services. For example, most countries provide more generous perinatal leave benefits and more generous extended leave benefits if a child is critically ill than the US [16], thus enabling parents to spend more time with their infant in the NICU. In the first US trial of the mobile-enhanced FICare (mFICare) intervention, no overall group effect was found for the primary aim of weight gain. However, mFICare infants overall had fewer nosocomial infections, and infants whose parents had a parent mentor or participated in rounds had better weight gain, than FCC infants [17]. We report here our findings comparing the effects of FCC with mFICare on mothers’ mental health after their preterm infant’s NICU discharge. We hypothesized that parents who participated in mFICare would have fewer symptoms of post-traumatic stress disorder (PTSD) and depression after their preterm infant’s NICU discharge than parents who received usual FCC. We also examined mFICare program components to determine whether they had differential effects on parent PTSD and depression symptoms.

## Methods

This analysis of pre-defined secondary aims was part of a larger quasi-experimental, time-lagged non-randomized intervention trial of the mFICare intervention compared with FCC (NCT03418870; 01/02/2018) [18]. Briefly, we analyzed data from two prospectively enrolled sequential cohorts of infant/parent dyads from three sites in California. The first cohort received usual FCC and the second received mFICare. All three NICUs were regional centers providing care to infants from ethnically diverse urban and rural communities: a level IV NICU in a university health system, a level IV NICU in a free-standing children’s hospital, and a level III NICU in a community hospital. Two of the sites provided high-risk maternity care with NICUs serving both inborn and outborn neonates, whereas the third site provided care to outborn neonates only. The NICUs all provided FCC as their standard model of NICU care and encouraged 24/7 parental presence.

### Participants

Parents/primary caregivers of infants born ≤ 33 weeks gestation were invited to participate. Participants were excluded if: (1) the parent was not English literate, < 18 years of age, or had no smart phone or tablet access; or (2) the infant had a life-threatening congenital anomaly or was receiving palliative care. Parents received up to $50 in gift cards for completion of study surveys. The study was approved by the institutional review board at each site and written informed consent was obtained from all participants [18]. Due to the small number of fathers who enrolled in the study (n = 16) and provided data for the post-discharge mental health measures (n = 9), fathers were excluded from this analysis.

### Intervention

Parents of current and former NICU patients and NICU healthcare professionals were extensively involved in the design of the trial and the adaptation of the FICare intervention to the local settings. Parents also co-designed and pilot-tested a mobile app for parents. Details of the FCC and mFICare interventions are provided elsewhere [18]. Briefly, parents/infants enrolled in the FCC cohort received the usual FCC provided in that NICU, including a supportive physical and interpersonal environment to encourage parents to spend extended periods in the NICU with their infant. Participants also received instruction on using We3health™ Tracker, an app designed for the FCC cohort to document time spent with their infant, document learning needs and skills acquisition, and a free text and photo journal for capturing their experiences and feelings. Parents in the mFICare cohort received all the supports and services of the FCC cohort. In addition, they were offered parent group education classes 2–5 times per week; training, encouragement, and an expanded role in direct infant caregiving (excluding ventilation management, intravenous fluid or intravenous medication administration); participation in weekday rounds; peer mentorship and an expanded version of the We3health™ app designed for the mFICare group.

The first cohort of enrolled parents received usual FCC. Then recruitment was paused for mFICare training of NICU staff and parent mentors. Once approximately 80% of each NICU’s staff received training, the second cohort of parent/infant dyads was enrolled and received mFICare.

### Measures

The primary outcomes for this analysis were maternal symptoms of perinatal PTSD and depression measured at least three months after the infant’s NICU discharge. The Perinatal PTSD Questionnaire (PPQ) [19] was used to assess self-reported symptoms of PTSD related to the childbirth experience and postnatal period. The PPQ includes 14 questions scored 0–4, and scores of 19 or higher indicate clinically significant symptoms of PTSD. The PPQ has demonstrated good internal consistency and test-retest reliability [20] and convergent and discriminant validity [21].

Maternal depression was assessed by self-report using the Edinburgh Postnatal Depression Scale (EPDS) [22]. The EPDS has well-established reliability and validity and is the most recommended screening tool for postpartum depression [23]. The EPDS has 10 items, scored 0–3, with a total score range of 0–30. Scores of 10 to 12 indicate probable mild depression risk requiring monitoring and scores greater than 12 indicate major depressive disorder risk. We chose a cut off score of 10 or higher to capture the range of postpartum depression severity risk from mild to severe [24].

We examined four potential moderators of the intervention effect on PTSD or depression symptoms: GA at birth (i.e., did the intervention effect differ based on the infant’s GA at birth?); Infant age at enrollment (i.e., did the intervention effect differ based on when it was started after the baby’s birth?); Infant discharge on respiratory or feeding devices or monitoring equipment (i.e., did the intervention effect differ based on whether the infant was discharged on a medical device); and Parent stress (i.e., did the intervention effect differ based on the parents’ perceived NICU-related stress at study enrollment). The selected moderators have been shown in prior studies to influence PTSD or depression in mothers after their infant’s discharge from the NICU [25, 26]. Perceived NICU-related stress was measured at enrollment using the Parental Stressor Scale: NICU (PSS:NICU) [27]. It includes 46 self-report items measuring parental stress related to four domains of the NICU experience. Each item is rated from 1 (not stressful at all) to 5 (extremely stressful), with higher scores indicating higher levels of stress. The PSS:NICU demonstrates good validity and reliability for parents of infants in NICU settings [28].

Additional sample characteristics were obtained from the infant medical record and parents surveys. Parents’ perception of the family-centeredness of NICU care they received was assessed near the time of discharge with the Digi Family-Centered Care-Parent Version (DigiFCC-P); scores range 1–7 with high scores indicating high perceived quality of FCC [29].

### Statistical analysis

Analyses were performed using R v4.1 [30] and Stata v14.2 (StataCorp LLC, College Station, TX). Descriptive statistics included frequencies for categorical variables and means with standard deviations for continuous variables. Variables were assessed for normality and transformed as appropriate. Group differences were assessed using Chi-square tests for categorical variables and independent t-tests for continuous variables. P-values < 0.05 were considered statistically significant for group comparisons of sample characteristics.

We tested for associations between PPQ and EPDS scores (log-transformed) and intervention group using a linear regression model. We first tested for main effects of the intervention. We adjusted for additional covariates using a hybrid approach, forcing in site, and then using a backwards stepwise regression to identify demographic and clinical covariates that contributed p < .1 to the final model.

We then tested for moderating effects on the intervention with four factors: infant gestational age at birth, infant chronological age at study enrollment (log transformed), whether the infant was discharged on a respiratory or feeding device, and NICU-related stress level at study enrollment (log-transformed), controlling for additional covariates as described above. Finally, for factors that had an interaction with the intervention group (denoted intervention moderators) with p < .05, we conducted an exploratory per protocol analysis testing for an interaction between the moderator and each of the following mFICare intervention components over the course of the NICU hospitalization: whether the parent had a mentor; participated in at least 2 clinical rounds; attended at least 1 parent class; or logged into the We3health™ app at least 4 times. We report results meeting a nominal p < .05, as none reached Bonferroni-corrected significance.

## Results

### Sample characteristics

**Fig. 1:**
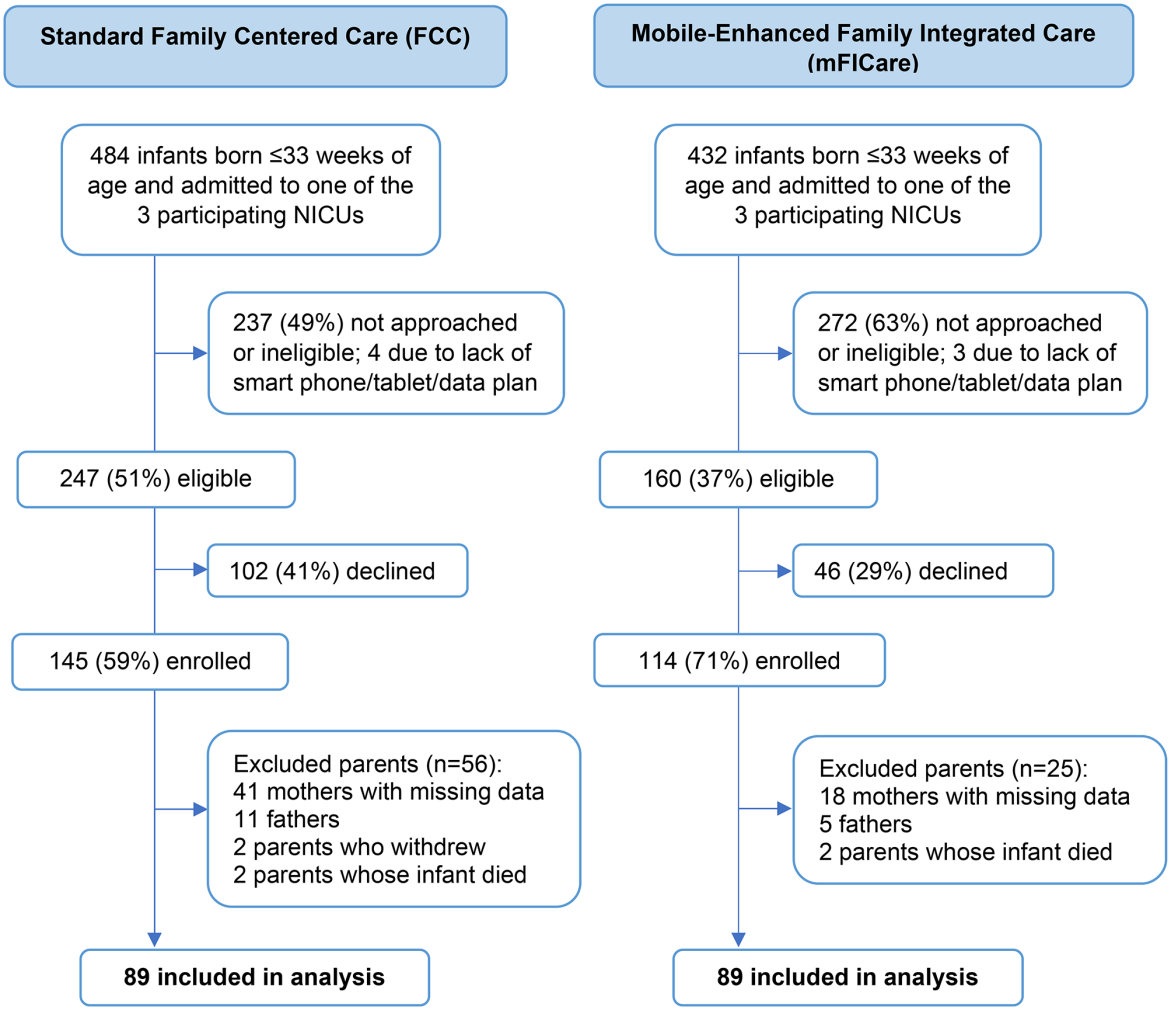
Participant flow diagram

Of the 237 mothers enrolled in the study between April, 2017 and June, 2020, 178 (75%; 89 FCC, 89 mFICare) completed PPQ and EPDS measures a mean of 4.2 (SD 1.9) months after their infant’s NICU discharge and were included in this analysis (Fig. 1).

Maternal characteristics are summarized in Table 1 and infant characteristics are summarized in Table 2. The only differences in sample characteristics by intervention group were that in the mFICare group multiple births were more common (21% vs. 9%, p = .02), fewer infants had nosocomial infections (6% vs. 15%, p = .05), and some of the mFICare group participated during the COVID-19 pandemic (21% vs. 0%, p < .001).

**Table 1:** Maternal characteristics by intervention group (n = 178)

Maternal characteristics	FCC (n = 89)	mFICare (n = 89)	P-value
***Care context***			
NICU Site, % (n)			0.21
Site A	37% (33)	48% (43)	
Site B	18% (16)	19% (17)	
Site C	45% (40)	33% (29)	
NICU stay during COVID-19 pandemic, % (n)	0% (0)	21% (19)	< 0.001
Mean miles from family home to hospital	55 (77)	50 (72)	0.66
Mean area deprivation index (ADI) of family home	41 (31)	34 (30)	0.16
***Maternal demographics***			
Mean age, years	29.8 (6.9)	31.7 (6.7)	0.07
Race/ethnicity, % (n)			0.29
Asian	20% (18)	17% (15)	
Black	13% (12)	14% (12)	
Hispanic/Latina	37% (33)	34% (30)	
White	27% (24)	25% (22)	
Other	2% (2)	10% (9)	
Post-secondary education, % (n)	76% (68)	81% (71)	0.49
Employed, % (n)	61% (54)	65% (57)	0.57
Primary language other than English, % (n)	24% (21)	20% (18)	0.61
Born outside the United States	20% (18)	23% (20)	0.71
***Maternal NICU experience measures***			
Stress (PSS:NICU score) at enrollment (median 2.5 weeks after NICU admission)	(n = 87)	(n = 85)	
Mean (SD)	2.3 (0.8)	2.3 (0.7)	0.87
Perception of family-centered care at discharge	(n = 84)	(n = 82)	
Mean (SD)	6.3 (0.9)	6.3 (0.8)	0.83
***Maternal mental health measures***			
PTSD (PPQ score) after discharge	(n = 88)	(n = 86)	
Mean (SD)	10.4 (10.8)	10.6 (9.3)	0.90
% (n) ≥ 19	18% (16)	17% (15)	0.90
Depression (EPDS score) after discharge	(n = 89)	(n = 89)	
Mean (SD)	6.0 (5.5)	6.0 (4.4)	0.98
% (n) ≥ 10	21% (19)	20% (18)	0.85
Both PTSD and depression symptoms	(n = 88)	(n = 86)	0.47
% (n) with PPQ ≥ 19 and EPDS ≥ 10	11% (10)	8% (7)	

Note: Data are presented as mean (SD) or % (n). P-values are for independent t-tests or chi-square tests, as appropriate. ADI = Area Deprivation Index – national rank (1 = least deprivation, 100 = most deprivation); PSS:NICU = Parental Stressor Scale: NICU; PTSD = Post-Traumatic Stress Disorder; PPQ = Perinatal Post-Traumatic Stress Disorder Questionnaire; EPDS = Edinburgh Postnatal Depression Scale

**Table 2:** Infant characteristics by intervention group (n = 178)

Infant characteristics	FCC (n = 89)	mFICare (n = 89)	P-value
***Birth characteristics***			
Mean gestational age, weeks	28.7 (2.7)	28.7 (2.5)	0.92
Mean birthweight, grams	1208 (485)	1180 (432)	0.68
Multiple birth, % (n)	9% (8)	21% (19)	**0.02**
Caesarean delivery, % (n)	60% (53)	63% (56)	0.64
Mean Apgar score 5 min after birth	6.9 (1.8)	6.8 (2.0)	0.69
***Clinical interventions during NICU stay***			
Ventilation, % (n)	56% (50)	58% (52)	0.76
Any surgeries, % (n)	25% (22)	24% (21)	0.83
Mean days on total parenteral nutrition	20.4 (23.6)	24.3 (29.6)	0.32
***Clinical diagnoses***			
Intraventricular hemorrhage, % (n)	18% (16)	21% (19)	0.57
Necrotizing enterocolitis, % (n)	9% (8)	10% (9)	0.80
Bronchopulmonary dysplasia, % (n)	19% (17)	20% (18)	0.85
Nosocomial infection, % (n)	15% (13)	6% (5)	**0.05**
Retinopathy of prematurity, % (n)	37% (33)	31% (28)	0.43
***Discharge characteristics***			
Discharged on human milk feeding, % (n)	67% (89)	57% (51)	0.16
Discharged on respiratory or feeding device, % (n)	27% (24)	34% (30)	0.33
Mean length of hospital stay, days	73.4 (49.0)	77.3 (41.5)	0.57

Note: Data are presented as mean (SD) or % (n). P-values are for independent t-tests or chi-square tests, as appropriate

### Measures of maternal mental health by intervention group

Measures of maternal mental health for each intervention group are shown in Table 1. There were no differences in PTSD or depression symptom scores after discharge between the mFICare and FCC groups (p = .47), with approximately 29% of the sample reporting clinically significant symptoms of PTSD, depression or both.

### Main intervention effects and moderators

As shown in Table 3, there were no main effects of the mFICare intervention on either maternal PPQ (p = .33) or EPDS scores (p = .45). Unsurprisingly, mothers’ level of NICU-related stress was significantly associated with both maternal PPQ (β = 1.05, 95% CI = 0.55 to 1.54, p < .001) and EPDS scores (β = 0.50, 95% CI = 0.10 to 0.90, p = .015).

**Fig. 2:**
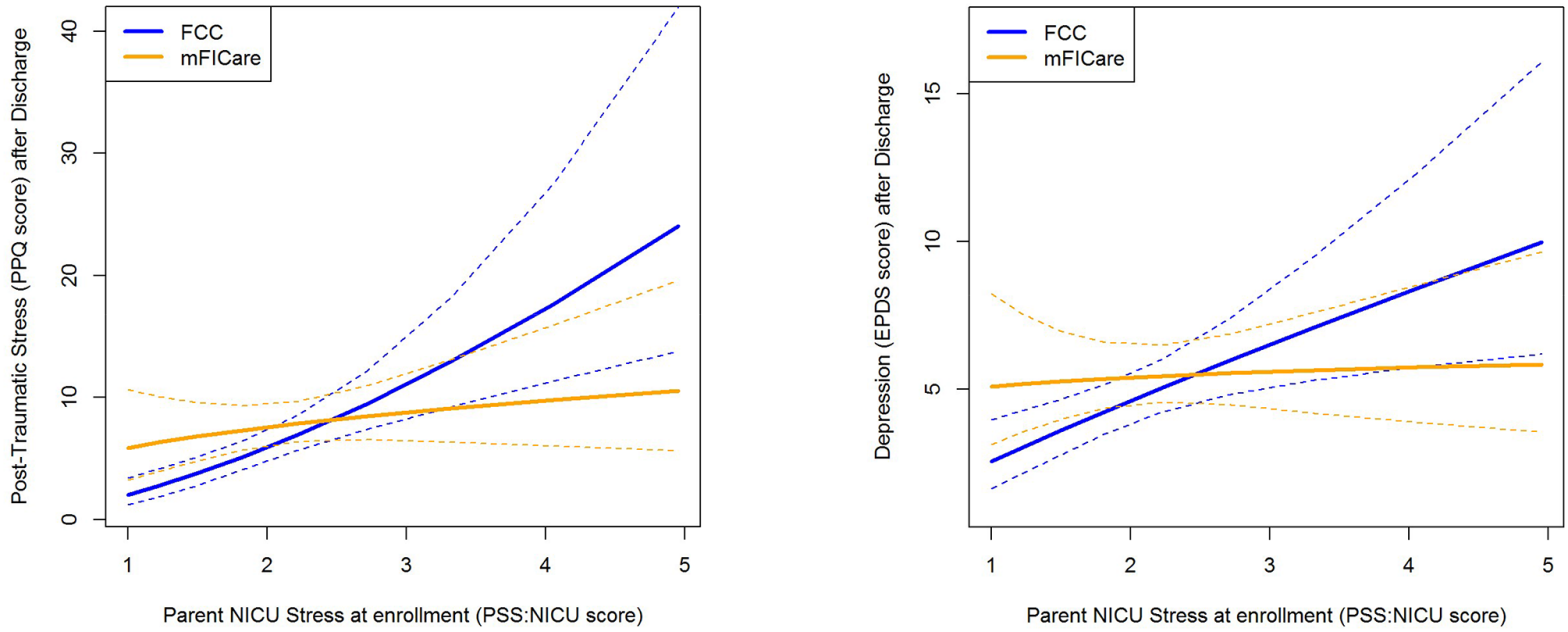
Interaction effects of parent NICU stress and mFICare intervention on PTSD symptoms (a) and depression (b) after NICU discharge. Dotted lines indicate the 95% confidence interval for each group. **a.** Mothers in the mFICare group with higher levels of NICU-related stress had lower post-discharge PPQ scores than similarly stressed mothers in the FCC group; mothers with lower levels of NICU-related stress had relatively few PTSD symptoms, regardless of intervention group (p = .012). **b.** A similar interaction pattern was found for maternal depression (EPDS) scores but did not reach statistical significance (p = .052)

Of the four potential moderators evaluated, there was no evidence that the intervention was differentially associated with the infant’s gestational age, infant age when mFICare was started, or whether the infant was discharged on a feeding and/or respiratory device (tested with an interaction effect, p > .05). However, we found a nominally significant interaction association between PSS:NICU scores and intervention group for PPQ (interaction β=-1.18, 95% CI: -2.10, -0.26; p = .012; Table 3). The best way to understand this differential association (in the context of the main effects of both of these covariates) is through visualization (Fig. 2a). Mothers in the mFICare group who experienced high NICU-related stress reported lower post-discharge PPQ and EPDS scores than similarly stressed mothers in the FCC group, whereas for mothers who experienced low NICU-related stress, there were no differences in post-discharge PPQ and EPDS scores. A similar interaction pattern was found for EPDS, but it did not reach statistical significance (interaction β=-0.764, 95% CI: -1.53, 0.006, p = .052; Table 3; Fig. 2b).

**Table 3:** Intent-to-treat analysis of the main and interaction effects of the mFICare intervention on maternal mental health

Models and statistics	PTSD (PPQ) models*	Depression (EPDS) models**
1. Main effects only		
Coefficients (95% CI) [p-value]		
a) mFICare (ref = FCC)	0.15 (-0.15, 0.45) [0.33]	0.10 (-0.16, 0.35) [0.45]
b) Stress (PSS:NICU score) at study enrollment (log)	1.05 (0.55, 1.54) [< 0.001]	0.50 (0.10, 0.90) [0.015]
Adjusted R^2^	0.221	0.063
Model F-statistic {df} [p-value]	8.41 {6,151} [< 0.001]	3.31 {5,166} [0.007]
2. Interaction effect		
Coefficients (95% CI) [p-value]		
a) mFICare (ref = FCC)	1.07 (0.39, 2.72) [0.007]	0.69 (0.04, 1.33) [0.037]
b) Stress (PSS:NICU score) at study enrollment (log)	1.55 (0.32, 4.91) [< 0.001]	0.85 (0.32, 1.39) [0.002]
c) Interaction of a) and b)	-1.18 (-2.10, -0.26) [0.012]	-0.76 (-1.53, 0.006) [0.052]
Adjusted R^2^	0.248	0.079
Model F-statistic {df} [p-value]	8.38 {7,150} [< 0.001]	3.45 {6,165} [0.003]

EPDS = Edinburgh Postnatal Depression Scale; FCC: family-centered care; PPQ = Perinatal Post-Traumatic Stress Disorder Questionnaire; PTSD = Post-Traumatic Stress Disorder; PSS:NICU: Parental Stressor Scale: NICU.

* PTSD model adjusted for site, length of hospital stay, 5-minute Apgar score, and mother’s perception of FCC quality at enrollment.

** Depression model adjusted for NICU site and infant gestational age.

### Per protocol analyses

**Fig. 3:**
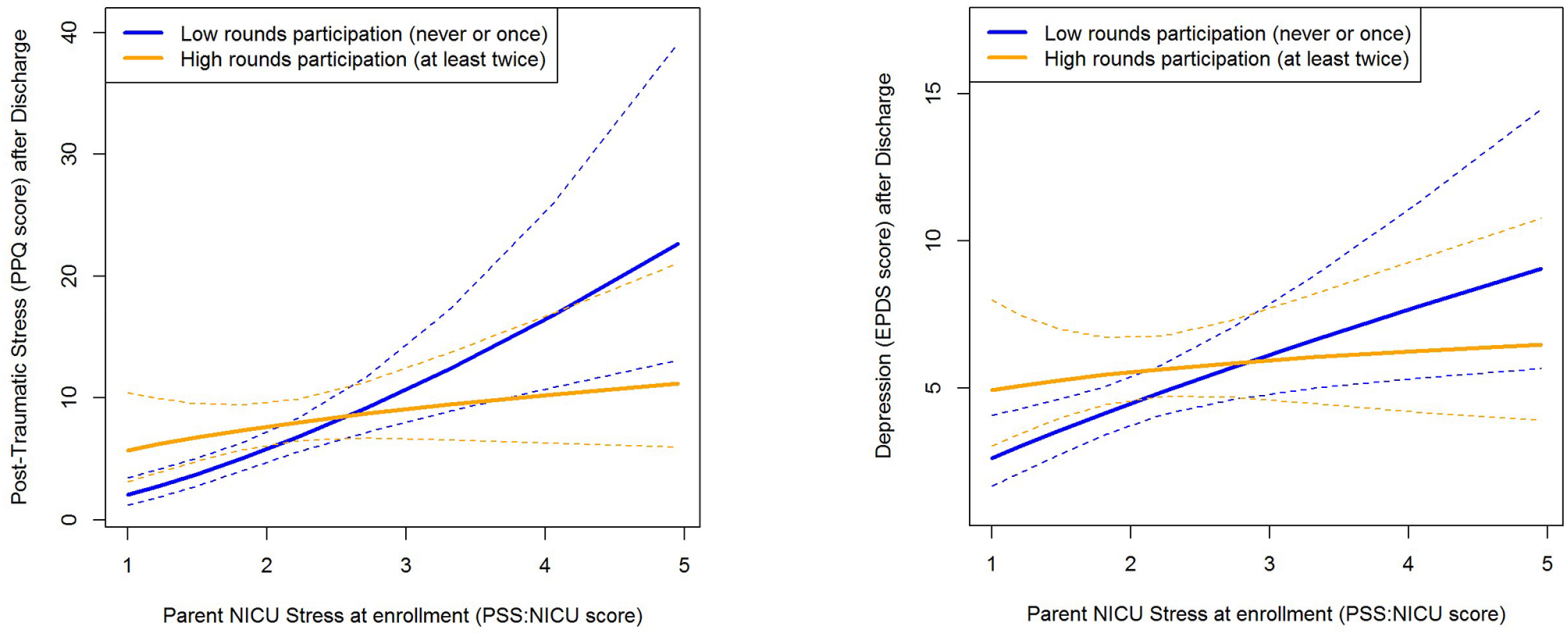
Interaction effect of parent NICU stress and attending mFICare clinical rounds on PTSD symptoms (a) and depression (b) after NICU discharge. Dotted lines indicate the 95% confidence interval for each group. **a.** Mothers with higher levels of NICU-related stress who participated in at least 2 daily rounds for their infant had lower post-discharge PPQ scores than similarly stressed mothers who did not; mothers with lower levels of NICU-related stress had relatively few PTSD symptoms, regardless of their participation in rounds (p = .022). **b.** A similar interaction pattern was found for maternal depression (EPDS) scores but did not reach statistical significance (p = .12)

Per protocol analyses were conducted to determine whether specific intervention components were differentially associated with maternal mental symptoms based on the mother’s level of NICU-related stress (i.e., an interaction effect with NICU-related stress). For mothers who experienced high levels of NICU-related stress, participating at least twice in clinical rounds was associated with fewer PTSD symptoms after discharge. However, for mothers who experienced low levels of NICU-related stress, there was no apparent association. This differential association (β=-1.08 [between clinical rounds and NICU-related stress]; 95% CI -1.99, -0.16; p = .022; Table 4) is best understood through visualization (Fig. 3a). A similar association trend was observed in the model predicting maternal depression, but it did not reach statistical significance (interaction β=-0.60; 95% CI -1.37, 0.16; p = .12 Fig. 3b).

**Fig. 4:**
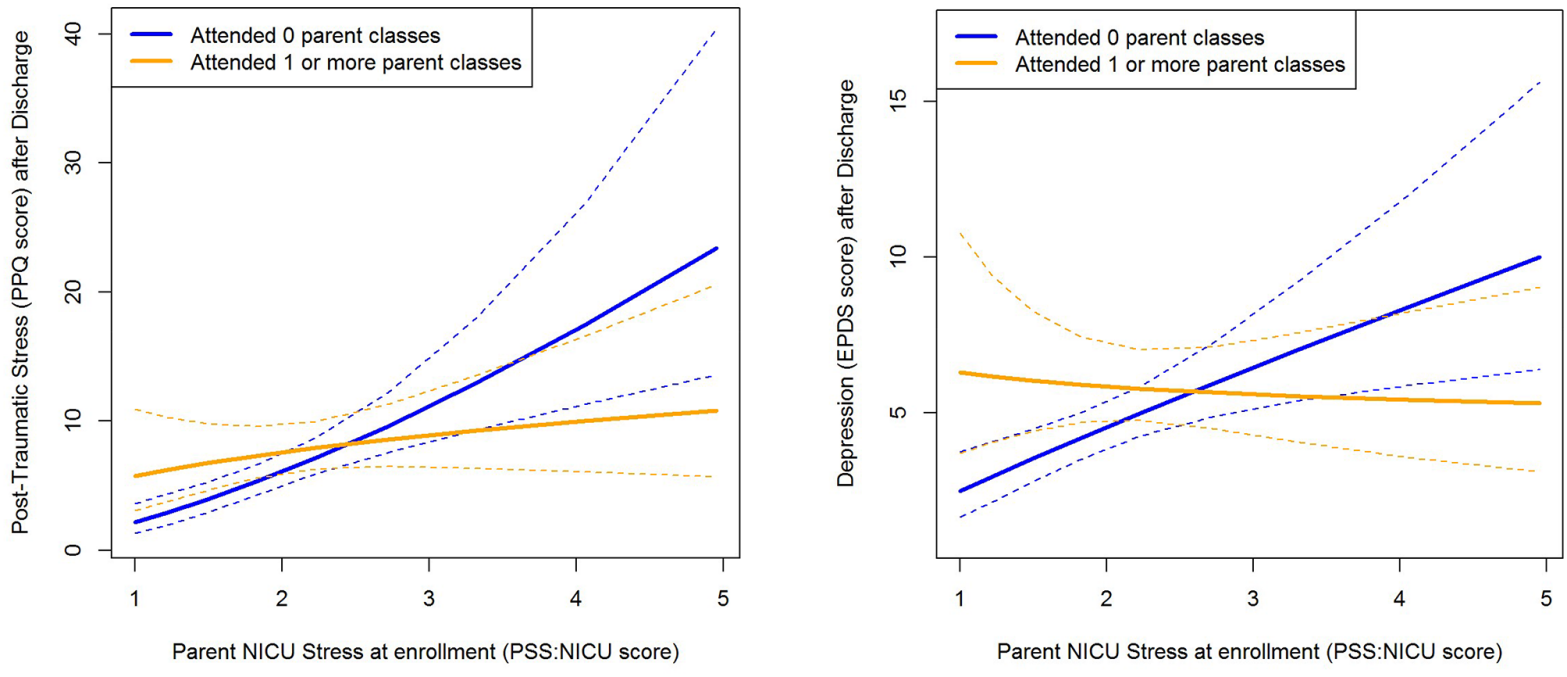
Interaction effect of mFICare parent classes and parent NICU stress and on PTSD symptoms (3a) and depression (3b) after NICU discharge. Dotted lines indicate the 95% confidence interval for each group. **a.** Mothers with higher levels of NICU-related stress who attended at least one parent class had lower post-discharge PPQ scores than similarly stressed mothers who did not attend classes; mothers with lower levels of NICU-related stress had relatively few PTSD symptoms, regardless of class attendance (p = .024). **b.** Mothers with higher levels of NICU-related stress who attended at least one parent class had lower post-discharge EPDS scores than similarly stressed mothers who did not attend classes; mothers with lower levels of NICU-related stress had relatively few depression symptoms, regardless of class attendance (p = .015)

Results were similar when examining if attending parental classes has a differential association with PTSD and depression symptoms after discharge. Among mothers who experienced high levels of NICU-related stress, those who attended at least one parent class experienced fewer PTSD symptoms (interaction β=-1.09, 95% CI -2.03, -0.16, p = .024) and fewer symptoms of depression (interaction β=-0.98, 95% CI -1.76, -0.20; p = .015) after discharge than similarly stressed mothers who did not attend parent classes. However, for mothers who had low levels of stress, there was no apparent association (Fig. 4a-b).

**Table 4:** Per protocol analysis of the main and interaction effects of the mFICare intervention components on maternal mental health

Models and statistics	PTSD (PPQ) models*	Depression (EPDS) models**
***Rounds participation***		
1. Main effects		
Coefficients (95% CI) [p-value]		
a) Rounds participation (ref = low)	0.19 (-0.11, 0.49) [0.22]	0.16 (-0.09, 0.42) [0.20]
b) Stress at study enrollment (PSS:NICU, score, log)	1.05 (0.56, 1.55) [< 0.001]	0.50 (0.10, 0.90) [0.015]
Adjusted R^2^	0.223	0.069
Model F-statistic {df} [p-value]	8.53 {6,151} [< 0.001]	3.55 {5,166} [0.005]
2. Interaction effect		
Coefficients (95% CI) [p-value]		
a) Rounds participation (ref = low)	1.02 (0.25, 1.80) [0.010]	0.63 (-0.02, 1.28) [0.12]
b) Stress at study enrollment (PSS:NICU, score, log)	1.50 (0.88, 2.12) [< 0.001]	0.77 (0.24, 1.30) [0.004]
c) Interaction of a) and b)	-1.08 (-2.00, -0.16) [0.022]	-0.60 (-1.37, 0.17) [0.12]
Adjusted R^2^	0.245	0.077
Model F-statistic {df} [p-value]	8.28 {7,150} [< 0.001]	3.38 {6,165} [0.004]
***Parent class attendance***		
1. Main effects		
Coefficients (95% CI) [p-value]		
a) Class attendance (ref = no)	0.12 (-0.19, 0.42) [0.46]	0.16 (-0.10, 0.42) [0.22]
b) Stress at study enrollment (PSS:NICU, score, log)	1.05 (0.56, 1.55) [< 0.001]	0.49 (0.09, 0.89) [0.017]
Adjusted R^2^	0.219	0.069
Model F-statistic {df} [p-value]	8.31 {6,151} [< 0.001]	3.52 {5,166} [0.005]
2. Interaction effect		
Coefficients (95% CI) [p-value]		
a) Class attendance (ref = no)	0.98 (0.17, 1.78) [0.018]	0.93 (0.26, 1.60) [0.007]
b) Stress at study enrollment (PSS:NICU, score, log)	1.48 (0.87, 2.10) [< 0.001]	0.87 (0.37, 1.37) [< 0.001]
c) Interaction of a) and b)	-1.09 (-2.03, -0.15) [0.024]	-0.98 (-1.76, -0.19) [0.015]
Adjusted R^2^	0.240	0.096
Model F-statistic {df} [p-value]	8.07 {7,150} [< 0.001]	4.02 {6,165} [< 0.001]

EPDS = Edinburgh Postnatal Depression Scale; PPQ = Perinatal Post-Traumatic Stress Disorder Questionnaire; PTSD = Post-Traumatic Stress Disorder; PSS:NICU = Parental Stressor Scale: NICU.

* PTSD model adjusted for site, length of hospital stay, 5-minute Apgar score, and mother’s perception of FCC quality at enrollment.

** Depression model adjusted for NICU site and infant gestational age.

Note: Parent mentorship and We3health™ app usage were unrelated to maternal health outcomes (data not shown).

## Discussion

Worldwide, approximately 25% of NICU parents report clinically significant PTSD symptoms [31] and up to 40% report clinically significant depression [32] in the months following NICU discharge. These estimates do not account for the model of NICU care received. In our study, around 1 in 5 mothers of preterm infants in NICUs providing either FCC or mFICare experienced clinically significant symptoms of PTSD or depression approximately 4 months after their infant’s discharge. Contrary to our hypothesis, our intention-to-treat analyses showed no overall differences in PTSD or depression symptoms between the two intervention groups. However, we found an interaction with NICU-related stress. Our findings suggest that mFICare may be more effective in preventing clinically significant PTSD symptoms than usual FCC alone for mothers experiencing high levels of NICU-related stress. Mothers experiencing low levels of NICU-related stress had relatively few PTSD or depression symptoms, regardless of intervention group. We speculate that the usual FCC practices at the three study sites, although diverse in practice, were sufficient to buffer the risk of adverse post-discharge mental health in mothers with low levels of NICU-related stress, but insufficient for mothers with high levels of NICU-related stress.

Previous studies have tended to focus on specific parent-focused therapies, for example trauma-focused therapy for PTSD [33], or mother-infant interaction or behavioral therapies for depression [34, 35]. A large 15-country study found a strong inverse association between parental perceptions of the level of FCC provided during the NICU stay and depression symptoms 4 months after discharge [36]. Our findings build on previous research and suggest that the mFICare model of NICU caregiving is particularly important for mothers experiencing high NICU-related stress, and that its specific components of participation in clinical rounds and parent group classes may be protective for these mothers in reducing clinically significant post-traumatic stress and depression symptoms after NICU discharge. Based on previous research, active participation by parents and shared decision-making during clinical rounds builds trust between parents and the clinical team, improves parental knowledge and is empowering [37]. We speculate that these positive short-term outcomes have long-term benefits in parental competence, confidence and mental health after discharge. The group learning and peer-support occurring during the mFICare parent classes lead to greater knowledge, social support and empowerment that are similarly protective [38, 39]. Further studies will be needed to examine the main and interaction effects of active parental participation in clinical rounds, group classes and peer-mentoring.

It is now widely recognized that comprehensive perinatal care must include preventative parental mental health services. For example, the California Law, Assembly Bill (AB) 3032-Maternal Mental Health Conditions Education, Early Diagnosis, and Treatment Act [40], required acute care hospitals to develop and implement a program to provide education and information to health care professionals and patients about maternal mental health conditions as of January 1, 2020 and extended coverage for postpartum mental health conditions to one year after birth. Our findings are a call to action to researchers and clinicians to incorporate standard assessment, prevention and treatment of parental mental health into routine NICU care [41]. Standardization and clinical implementation of validated measures of family exposure to FCC and mFICare intervention components and family perceptions of family-centeredness of services and involvement in infant caregiving and shared decision-making are also needed to evaluate fidelity to the interventions and relative impact on mental health outcomes. Further research is needed to improve FCC and mFICare delivery by NICU staff and to address parental financial or social barriers to participation in FCC or mFICare. More longitudinal studies are needed to investigate the impact of family-focused NICU interventions on post-discharge parental mental health and infant outcomes [15].

Our study had several limitations in design and implementation, including selection bias because of the non-random design, unmeasured participation of families in the usual FCC group and incomplete uptake of the mFICare intervention by all mothers in that group. Selection bias may have also affected recruitment. We note that 59% of eligible FCC participants enrolled and 71% of mFICare participants enrolled; nevertheless, there were few differences in characteristics of the two groups. Timing of the intervention may be important and affected by enrollment later in the admission. Other limitations include mFICare intervention curtailment by the COVID-19 pandemic, further reducing intervention dose for some participants, and the exclusion of non-English-speaking families, limiting generalizability. Strengths of the study include increased generalizability of the findings because of the racially and ethnically diverse participant families as well as populations served by the participating NICUs and use of mobile app technology to increase intervention access.

In conclusion, the mFICare model was not associated with fewer mental health symptoms for mothers than the FCC model overall, but it was associated with fewer mental health symptoms for those experiencing higher levels of stress during the NICU stay. Further research on mFICare is urgently needed to address the prevalent and persistent mental health symptoms experienced by parents of preterm infants.

## Data Availability

Deidentified data will be shared upon reasonable request directed to Linda S. Franck (linda.franck@ucsf.edu) from qualified investigators beginning 6 months and ending 5 years after publication.

## List of abbreviations

EPDS: Edinburgh Postnatal Depression Scale
FCC: Family-Centered Care
FICare: Family Integrated Care
GA: Gestational Age
mFICare: mobile-enhanced Family Integrated Care
NICU: Neonatal Intensive Care Unit
PPQ: Perinatal PTSD Questionnaire
PTSD: Post-Traumatic Stress Disorder
US: United States
